# Validity of Machine Learning in Predicting Giant Cell Arteritis Flare After Glucocorticoids Tapering

**DOI:** 10.3389/fimmu.2022.860877

**Published:** 2022-04-05

**Authors:** Vincenzo Venerito, Giacomo Emmi, Luca Cantarini, Pietro Leccese, Marco Fornaro, Claudia Fabiani, Nancy Lascaro, Laura Coladonato, Irene Mattioli, Giulia Righetti, Danilo Malandrino, Sabina Tangaro, Adalgisa Palermo, Maria Letizia Urban, Edoardo Conticini, Bruno Frediani, Florenzo Iannone, Giuseppe Lopalco

**Affiliations:** ^1^ Department of Emergency and Organ Transplantation, Rheumatology Unit, University of Bari, Bari, Italy; ^2^ Department of Experimental and Clinical Medicine, University of Florence, Florence, Italy; ^3^ Research Centre of Systemic Autoinflammatory Diseases, Behçet’s Disease Clinic and Rheumatology-Ophthalmology Collaborative Uveitis Centre, Department of Medical Sciences, Surgery and Neurosciences, University of Siena, Siena, Italy; ^4^ Rheumatology Department of Lucania, San Carlo Hospital of Potenza, Potenza, Italy; ^5^ Ophthalmology Unit, Department of Medicine, Surgery and Neuroscience, University of Siena, Siena, Italy; ^6^ Dipartimento di Scienze del Suolo, della Pianta e degli Alimenti, University of Bari “Aldo Moro”, Bari, Italy; ^7^ Istituto Nazionale di Fisica Nucleare - Sezione di Bari, Bari, Italy

**Keywords:** giant cell (temporal) arteritis, glucocorticoids, machine learning, algorithm, precision medicine

## Abstract

**Background:**

Inferential statistical methods failed in identifying reliable biomarkers and risk factors for relapsing giant cell arteritis (GCA) after glucocorticoids (GCs) tapering. A ML approach allows to handle complex non-linear relationships between patient attributes that are hard to model with traditional statistical methods, merging them to output a forecast or a probability for a given outcome.

**Objective:**

The objective of the study was to assess whether ML algorithms can predict GCA relapse after GCs tapering.

**Methods:**

GCA patients who underwent GCs therapy and regular follow-up visits for at least 12 months, were retrospectively analyzed and used for implementing 3 ML algorithms, namely, Logistic Regression (LR), Decision Tree (DT), and Random Forest (RF). The outcome of interest was disease relapse within 3 months during GCs tapering. After a ML variable selection method, based on a XGBoost wrapper, an attribute core set was used to train and test each algorithm using 5-fold cross-validation. The performance of each algorithm in both phases was assessed in terms of accuracy and area under receiver operating characteristic curve (AUROC).

**Results:**

The dataset consisted of 107 GCA patients (73 women, 68.2%) with mean age ( ± SD) 74.1 ( ± 8.5) years at presentation. GCA flare occurred in 40/107 patients (37.4%) within 3 months after GCs tapering. As a result of ML wrapper, the attribute core set with the least number of variables used for algorithm training included presence/absence of diabetes mellitus and concomitant polymyalgia rheumatica as well as erythrocyte sedimentation rate level at GCs baseline. RF showed the best performance, being significantly superior to other algorithms in accuracy (RF 71.4% vs LR 70.4% vs DT 62.9%). Consistently, RF precision (72.1%) was significantly greater than those of LR (62.6%) and DT (50.8%). Conversely, LR was superior to RF and DT in recall (RF 60% vs LR 62.5% vs DT 47.5%). Moreover, RF AUROC (0.76) was more significant compared to LR (0.73) and DT (0.65).

**Conclusions:**

RF algorithm can predict GCA relapse after GCs tapering with sufficient accuracy. To date, this is one of the most accurate predictive modelings for such outcome. This ML method represents a reproducible tool, capable of supporting clinicians in GCA patient management.

## Introduction

Giant Cell Arteritis (GCA) is the most common systemic vasculitis worldwide (1), and glucocorticoids (GCs) have been considered for several decades the mainstay of treatment. The symptoms of GCA should respond promptly to high-dose GCs following the resolution of the inflammatory response (2). To prevent glucocorticoid-related side effects deriving from prolonged courses of high-dose steroid therapy, GCs gradual tapering should be started without clinical symptoms, signs, and laboratory abnormalities suggestive of active disease. Unfortunately, many patients may experience a disease flare during tapering, requiring higher doses and the initiation of a GCs-sparing agent such as methotrexate or tocilizumab. To date, predictors of GCA relapse upon GCs treatment tapering are lacking. According to the 2018 EULAR recommendations for the management of large vessel vasculitis (3), inferential statistical methods failed to identify reliable biomarkers and risk factors for disease flare. Indeed, Logistic Regression (LR) is not suitable for modeling non-linear relationships and might be inadequate to clearly describe the complex relationship between prognostic factors and remission for multifactorial and unclear mechanisms of a disease condition like GCA (4).

In this regard, machine learning (ML) is emerging as a promising tool for implementing complex multi-parametric decision algorithms (5–8). Non-linear ML approaches such as tree-based algorithms, may allow to better handle complex non-linear associations between patient attributes that are hard to model with LR, merging them to output a forecast or a probability for a given outcome (4). ML can represent a step towards precision medicine in rheumatology, improving patient profiling and treatment personalization. Supervised ML algorithms have proven effective in predicting treatment responses and disease progression in patients with rheumatic diseases and grading synovitis in histopathological photomicrographs (4, 9). The early recognition of those more likely to experience a GCA flare may help clinicians schedule a personalized follow-up strategy and optimize GC treatment management.

Here we investigate whether an explainable ML approach may be helpful to predict a GCA flare upon GC treatment tapering.

## Materials and Methods

### Data Gathering

Patients with classified GCA according to the 1990 ACR criteria (10) referred to four Italian tertiary centers (Bari, Firenze, Potenza, Siena) for new-onset disease and treated with GCs from September 2018 to December 2020 were included in the analysis. Demographic (age and gender), laboratory (Erythrocyte Sedimentation Rate [ESR], C Reactive Protein [CRP] serum level) and clinical characteristics at presentation were retrospectively gathered, namely, disease duration in weeks of either fever, polymyalgia rheumatica (PMR), headache, jaw claudication, visual abnormalities aortic aneurysms, typical temporal artery biopsy, diabetes mellitus (DM), and cardiovascular comorbidities. We also kept track of imaging studies and biopsy procedures carried out on our patients. GCs induction dose was administered according to the judgement of the clinician, and treatment tapering was managed according to the 2008 EULAR recommendations and the Royal College of Physician guidelines (2, 11), aiming at a prednisone equivalent dose from 10 to 15 mg/day at 3 months after resolution of symptoms and laboratory abnormalities. Furthermore, we recorded the flare rate at three months from GCs tapering. The study was approved and reviewed by the local Ethical Committee (GISEA registry, IRB approval n. DG-624, Azienda Ospedaliera Universitaria di Bari; ClinicalTrial.Gov NCT01543594). This study followed the STARD guidelines and the TRIPOD statement. All patients provided written informed consent.

### Outcome of Interest

The predictive modeling analysis aimed to forecast the probability of GCA flare at 3 months following GCs tapering, represented as a Boolean variable. Flare was considered as the recurrence of signs or symptoms of GCA. We kept track of the first flare following GCs tapering only.

### Attributes Selection

The attribute core set used to train the algorithms was determined using a cross-validated recursive feature elimination wrapper based on a decision tree algorithm with extreme gradient boosting (XGBoost) described elsewhere (4). Briefly, this algorithm automatically selects the best number of features among all the gathered attributes based on their importance for predictions of the given outcome (**Supplementary Material**). We point out that, by design, the feature selection algorithm never relies on validation data to achieve this result. We repeated this process five times using different random seeds to check feature stability. The most often selected attributes were considered part of the final attribute core set (**Supplementary Material**).

### Algorithm’s Training and Validation

The analysis was implemented in a Python 3.8 environment using scikit-learn (ver. 0.22.1) and XGBoost (ver. 1.1.0) libraries (12). After z-score normalization, we ran a Bayesian ridge conditional imputation for missing data. Three different linear and non-linear classifiers were trained and validated with 5-fold cross-validation for predicting GCA flare. For further details about cross-validation see **Supplementary Material**. For linear modeling, an LR was used; for non-linear modeling, decision tree-based algorithms of growing complexity, namely, simple Decision Tree (DT) and Random Forest (RF) (13), were tested. A repeated grid search with cross-validation was used for optimal hyperparameter tuning to maximize the performance of the classifiers (**Supplementary Material**) (12). For each classifier, we plotted ROC curves, and then AUROC was determined. Then, based on the optimal probability cut-off (Youden’s Index (14)), the performance of the classifiers was compared with the following metrics after 5-fold cross-validation:


$$\text{Accuracy}=\frac{truepositives+truenegatives}{truepositives+truenegatives+falsepositives+falsenegatives}$$

$$\text{Recall}=\frac{truepositives}{truepositives+falsepositives}$$

$$\text{Precision}=\frac{truepositives}{truepositives+falsepositives}$$

### Probability Calibration

A classification model generally forecasts a binary outcome for a given observation and class. In the process of predicting, a model may output the probability of an observation belonging to each possible class (4). This case provides some flexibility in the way predictions are interpreted and presented, allowing the choice of a threshold, as mentioned above, Youden’s index. For a model to be reliable, the estimated class probabilities should reflect the true underlying probability of the sample. A diagnostic calibration curve for the candidate best classifier was also plotted to check these assumptions and, consequently, isotonic calibration was carried out.

## Results

**Figure 1:**
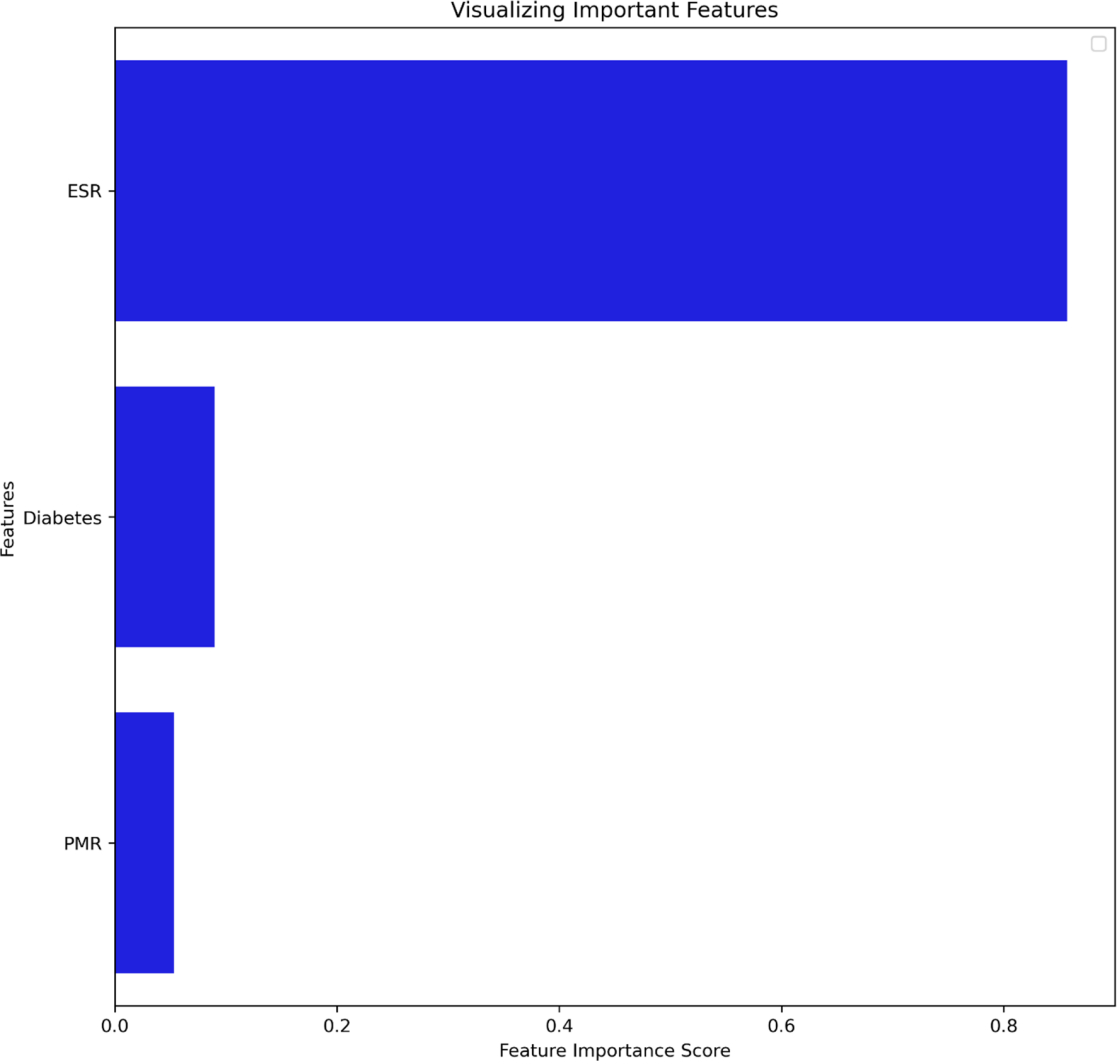
Attribute core set used for training and validation of the algorithms ranked for feature importance score. DM, Diabetes mellitus; ESR, Erythrocyte Sedimentation Rate; PMR, Polymyalgia Rheumatica.

**Figure 2:**
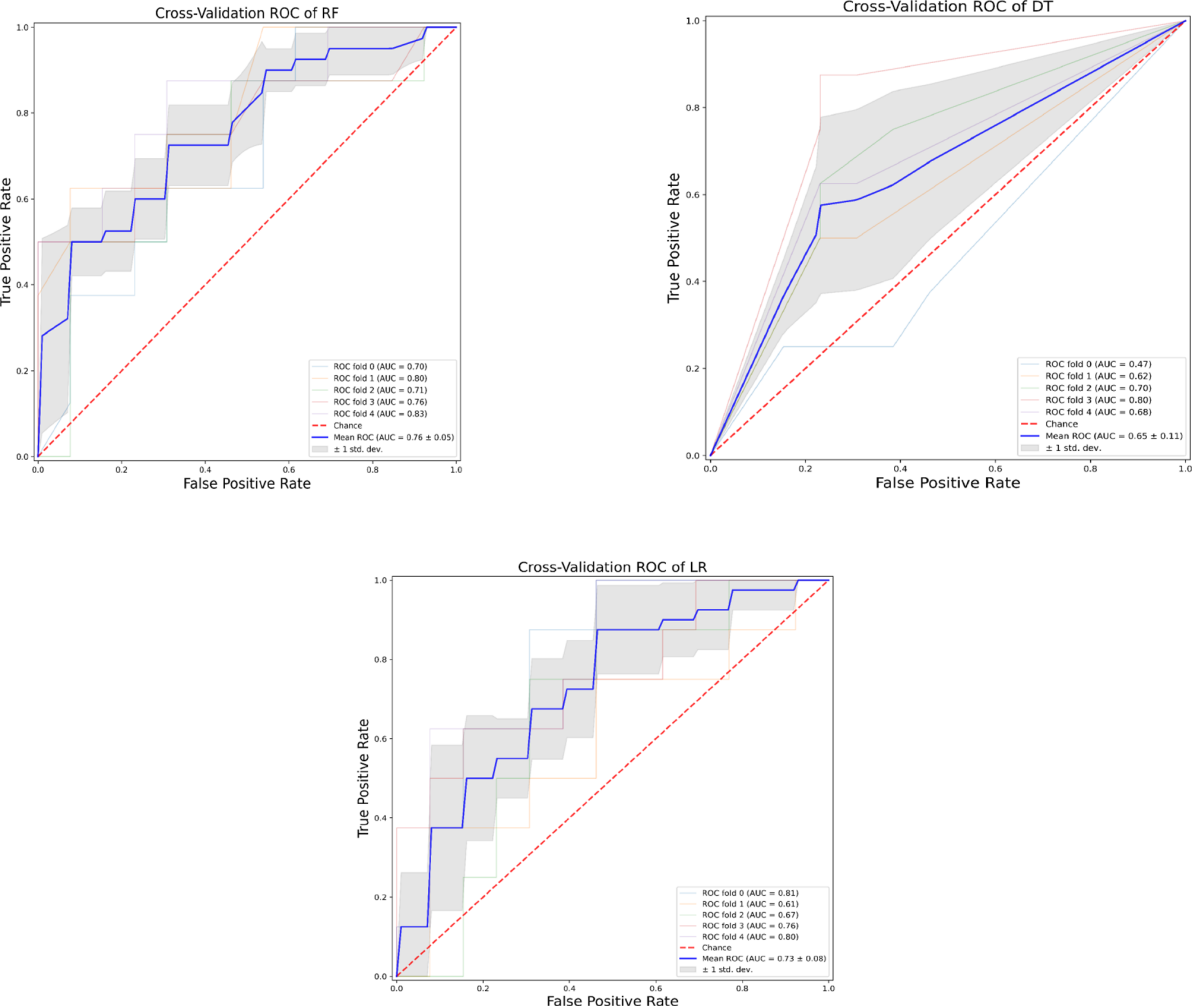
Receiver operating characteristic curves of the assessed algorithms. AUROC, Area under the receiver operating characteristic curve; DT, Decision tree; LR, Logistic Regression; RF, Random Forest.

**Figure 3:**
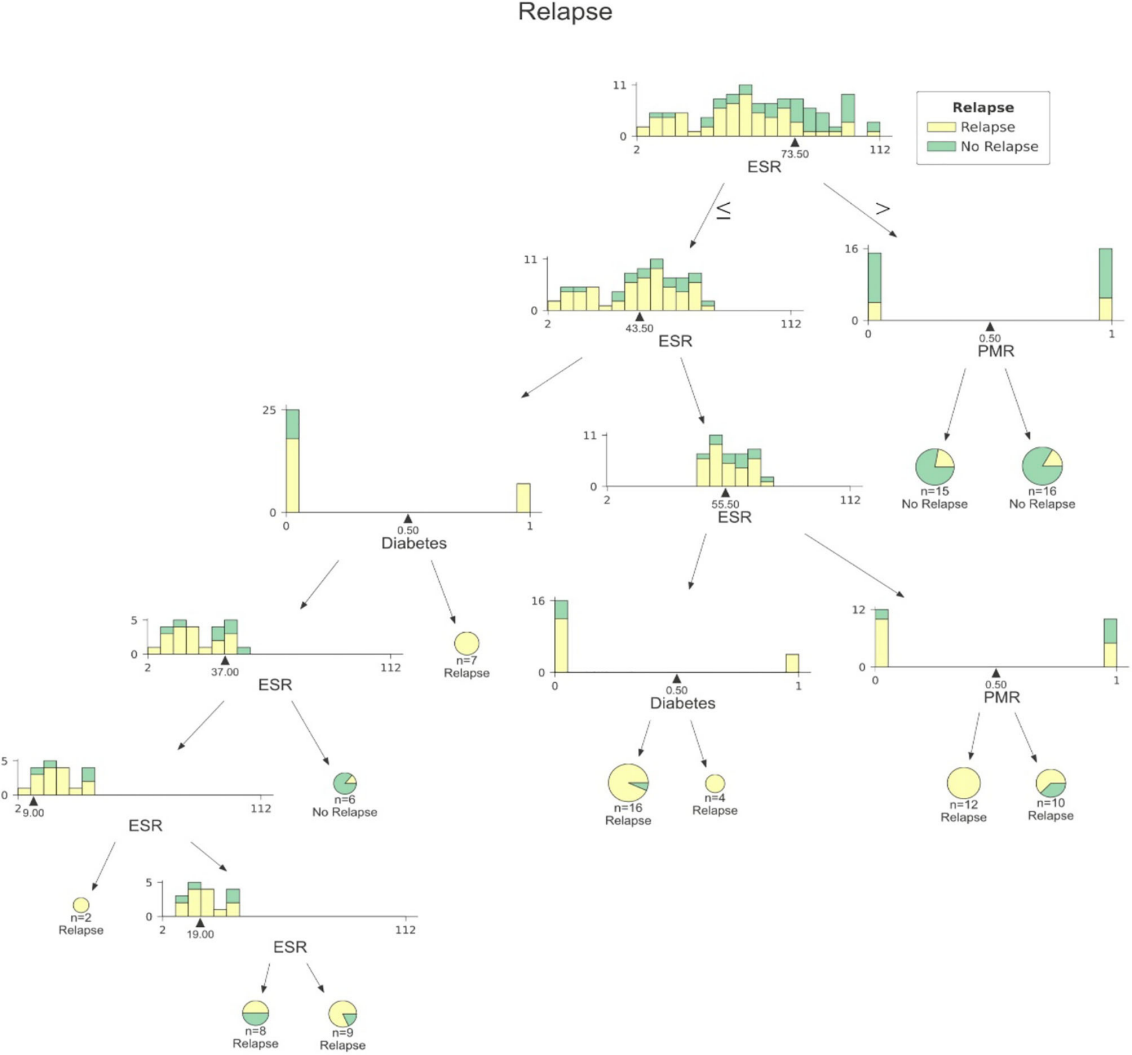
A sample decision tree among those included into RF. At each node data are split according to ESR, presence/absence DM or presence/absence of PMR. DM, Diabetes mellitus; ESR, Erythrocyte Sedimentation Rate; PMR, Polymyalgia Rheumatica.

**Figure 4:**
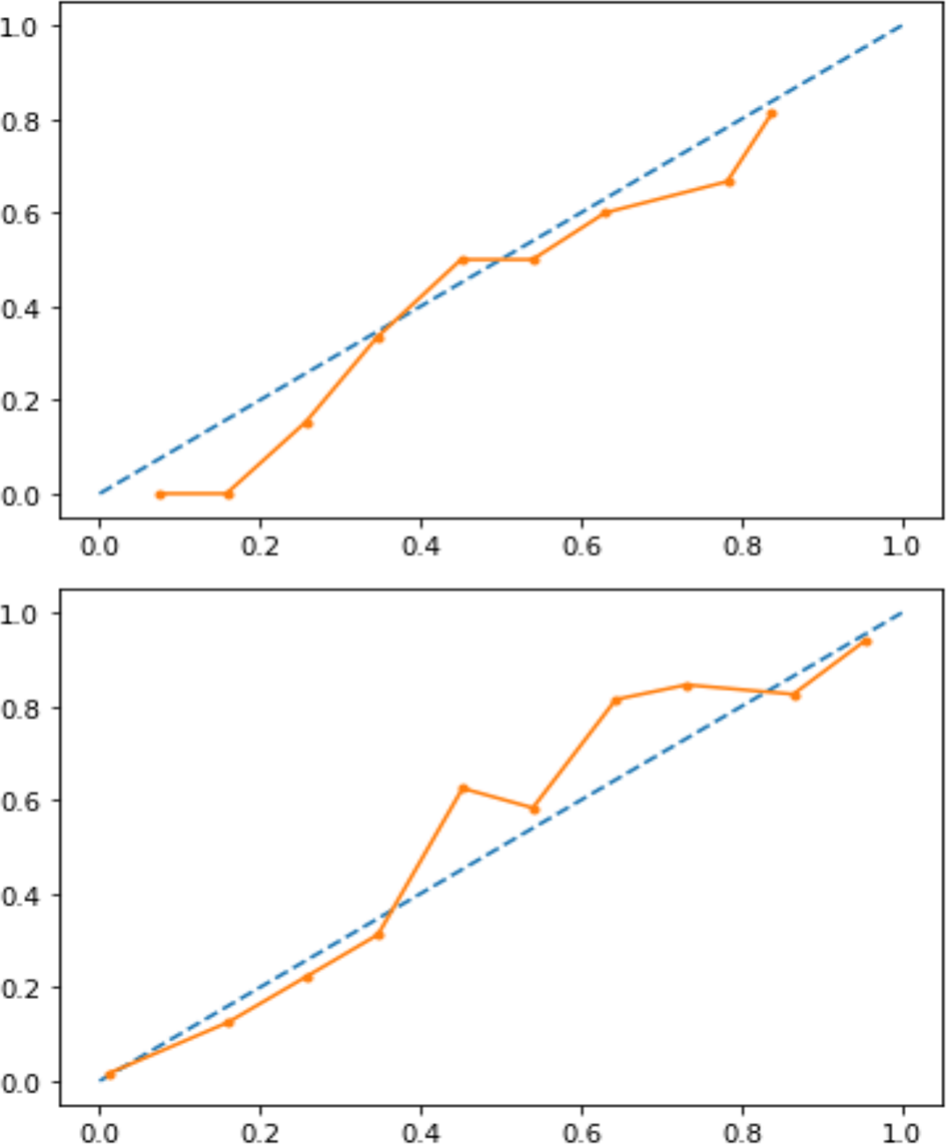
Calibration curve before (upper panel) and after (lower panel) isotonic calibration. After calibration, GCA flare roughly happened with an observed relative frequency (dotted line) consistent with the forecast value (solid orange line), showing an acceptable calibration.

Our analysis included 107 patients with classified GCA (73 women, 68.2%) with mean age ( ± SD) of 74.1 ± 8.5 years and mean symptom duration of 17.7 ± 37.5 weeks who underwent GCs treatment with a mean dose of 27.7 ± 17.6 prednisone dose mg/daily. Headache was the most prevalent symptom at GCs baseline, being present in 82/107 patients (76.6%); 33 out of 107 (30.8%) complained of ophthalmologic symptoms, whereas PMR was diagnosed in 58 of them (54.2%). Temporal artery abnormalities were detected at the clinical exam in 33/105 patients (31.4%), in two patients, information about signs of superficial artery inflammation was missing. A “halo” sign was seen in 67 of the 78 patients (85.9%) who underwent temporal artery ultrasound examination. Temporal artery biopsy was carried out in 27 patients and was positive in 15 of them (55.6%). Mean Body Mass Index (BMI) was 26.1 ± 4.2, whereas 21/107 (19.6%) had diabetes mellitus. Mean ESR and CRP at GCs baseline was 70.6 ± 62.2 mm/h and 50.5 ± 80.8 mg/L, respectively (**Table 1**). GCA flare occurred in 40/107 patients (37.4%) within 3 months from GCs tapering. At that time, prednisone mean dose was 10.5 ± 4.1 mg/daily, whereas mean ESR and CRP were 46.4 ± 22.1 mm/h and 26.7 ± 5.9 mg/L, respectively. PMR (27/40 patients, 67.5%) followed by headache (10/40, 25%) were the most frequent symptoms at disease flare (**Table 1**). Three out of 40 patients complained of ophthalmologic symptoms (7.5%). The attribute core set with the highest accuracy retrieved from the feature selection wrapper consisted of 3 attributes, namely ESR and presence/absence of DM and PMR (both represented as Boolean variables, **Figure 1**). RF showed the best performance, being significantly superior to other algorithms in accuracy (RF 71.4% vs LR 70.4% vs DT 62.9%). The out-of-bag error for RF was 0.714. Consistently, RF precision (72.1%) was significantly greater than those of LR (62.6%) and DT (50.8%). Conversely, LR was superior to RF and DT in recall (RF 60% vs LR 62.5% vs DT 47.5%). Coefficients for LR have been provided in the **Supplementary Material**. In **Figure 2**, ROC curves for analyzed algorithms were plotted. RF AUROC (0.76) was more significant compared to LR (0.73) and DT (0.65). Since *k*-fold cross-validation produces a sampling from the error distribution of the model, it allows computing the expected value of the metrics and the standard deviation on the metrics. We ran paired two-tailed t-tests to confirm that the differences in results between different models were statistically significant. Each possible pairing of metrics always resulted in statistically significant differences between the models (p <0.0001 in all mentioned cases). For further details, see **Table 2**. In **Figure 3**, a diagnostic calibration has been plotted for RF before and after isotonic calibration. GCA flare roughly happened with an observed relative frequency consistent with the forecast value, showing a suitable calibration curve. We expect the match between predicted frequencies and observed frequencies to increase with a larger dataset. The RF is an ensemble of decision trees. For the sake of clarity, **Figure 4** reports one of the decision trees used by RF for GCA flare prediction based on the attribute core. We also reported the importance of each attribute within the core set giving a glimpse of the attributes to check for algorithm implementation. The importance of ESR was greater than the presence/absence of DM and PMR (**Figure 1**).

**Table 1:** Cohort characteristics.

Cohort Characteristics	BASELINE		RELAPSE	
	Av. Obs.		Av. Obs	
Female, n (%)	107	73 (68.2)		
Age, mean (SD)	107	74.1 (8.5)		
Disease duration, weeks, mean (SD)	107	17.7 (37.5)		
BMI, mean (SD)	107	26.1 (4.2)		
**Type II DM, n (%)**	107	21 (19.6)		
Cardiovascular diseases, n (%)	104	68 (65.4)		
Temporal artery abnormalities, n (%)	105	33 (31.4)		
Positive temporal artery biopsy, n (%)	27	15 (55.6)		
Halo sign, n (%)	78	67 (15.9)		
**ESR, mm/h, mean (SD)**	101	56.3 (27.3)	40	10.5 (4.1)
CRP, mg/l, mean (SD)	102	50.5 (80.8)	38	46.4 (22.1)
Fever (>38°C), n (%)	107	41 (38.3)	40	2 (5)
Headache, n (%)	107	82 (76.6)	40	10 (25)
Ophthalmologic symptoms, n (%)	107	33 (30.8)	40	3 (7.5)
Weight loss (>2 kg), n (%)	103	32 (31.07)		
Jaw claudication, n (%)	107	36 (33.6)		
Aortic aneurysm, n (%)	106	3 (2.83)		
**Polymyalgia rheumatica symptoms, n (%)**	107	58 (54.2)	40	27 (67.5)
Glucocorticoid dose, mg PDN, mean (SD)	107	27.7 (17.6)	40	10.5 (4.1)
Time to remission, weeks, mean (SD)	57	3.8 (2.4)		
Relapse at three months for steroid tapering, n (%)	107	407 (37.4)		

CRP, C reactive protein; DM, Diabetes mellitus; ESR, Erythrocyte Sedimentation Rate; GCs, Glucocorticoid.

The wrapper algorithm automatically selected among all the gathered attributes at baseline the best number of features based on their importance for predictions of GCA flare. The attributes selected as the core set to train algorithms are all in bold.

**Table 2:** Performance of the ML algorithms.

	Cut-off	Accuracy (%)	SD	Recall (%)	SD	Precision (%)	SD	AUROC	SD
LR	0.52	70.4	0.11	62.5*	0.25*	62.6	0.08	0.73	0.08
RF	0.46*	71.4*	0.06*	60	0.14	72.1*	0.05*	0.76*	0.05*
DT	1	62.9	0.07	47.5	0.14	50.8	0.11	0.65	0.11

*p < 0.001.

AUROC, area under the receiver operating characteristic curve; DT, Decision tree; LR, Logistic Regression; RF, Random Forest.

## Discussion

Despite GCs treatment, several reports in the literature have shown that up to 68% of patients will have at least one relapse during GCA, thus increasing both the cumulative amount and duration of GCs therapy (15–17). Chronic use of GCs increases the risk of several comorbid conditions, namely, avascular necrosis, osteoporosis, fracture, infections, cardiovascular disease, and worsening pre-existing diabetes (18, 19). Determining which patients are at higher risk of relapse may help rheumatologists manage GCA patients better. ML algorithms have proven helpful at patient profiling both with supervised and unsupervised methods in several fields of medicine, including rheumatology. The tree-based approach is particularly intriguing for predictive modeling of complex diseases as non-linear conditions cannot be solved with LR since it has a linear decision surface. Linearly separable data is rarely found in a real-world scenario. Nevertheless, LR may account for the well-known advantage to provide an estimate of effect size as odds ratio, which is easily interpretable for clinicians in evaluating associations (5). On the other hand, considerable efforts in making ML explainable have been made, providing insights on the inner mechanism of tree-based algorithms, plotting feature importance and easy-to-read decision tree graphs (**Figures 1**, **3**) (20). The RF algorithms showed good accuracy in identifying those at risk for flare, being superior to traditional LR and DT. According to ML, the identikit of the GCA patient more likely to experience a flare at 3 months from GCs tapering has high ESR at baseline together with comorbid diabetes, and PMR. Due to its specific study design supporting predictive modeling at 3 months after GCs tapering than mere descriptive statistics during a certain follow-up period, it is hard to compare our data to those in literature. In our cohort, flares recurred in the 37.4% of our patients at 3 months after treatment tapering that is roughly comparable to data from Labarca and colleagues (16) who described flare in up to 50% of their cohort in the first year of treatment. Nevertheless, reported relapse rates may considerably vary as in the case of Kermani et al. (21) who described that only 24% (31/128) of their cohort experienced a first relapse by 12 months. In the interpretation of such results, one should consider that the frequency of relapses reported in observational cohorts of patients with GCA varies widely based on the study type and definition of relapse. In fact it should be noticed that in retrospective studies, at least a disease relapse was reported from 28 to 68% of patients (16, 17, 19, 22–24). Similarly to other studies (17), first relapses occurred when patients were receiving a mean prednisone dose of 10.75 ± 4.1 mg/daily. Previous studies showed that disease flares were rare on prednisone doses >20 mg/day (21). Kermani et al. (21) observed that 54% of the relapses occurred on a daily prednisone dose from 1 to 10 mg. Consistently to previous evidence, the candidate algorithm deemed valuable ESR as a marker of inflammatory burden. This is in line with the results of Restuccia et al. (17), who found that severity of inflammation, together with fever, were independent predictors of disease flare. Strikingly, in the latter paper, the most frequent clinical manifestation in patients who relapsed at a dosage of prednisone ≤ 10  mg/daily was PMR (49/73, 67.1%). PMR was also one of the most frequent symptoms at relapse (30 out of 59 flares, 51%) in the prospective cohort described by Kermani et al. (21). Not surprisingly, baseline PMR was one of the selected features capable of predicting flare in RF algorithm. Only a previous report from the Mayo Clinic GCA cohort (16) had shown diabetes as a predictor of disease flare. It is interesting to notice the high prevalence of diabetes in our cohort (21/107, 19.6%). In this regard it is worth mentioning that our cohort may have suffered from a selection bias due to the high comorbidity burden in patients from tertiary centers. On the other hand, the presence of diabetes among important attributes for prediction may reflect the attitude of clinicians to approach GCA patients with comorbid diabetes with lower GCs dose, which is likewise historically associated with short-term disease flare (16). Nevertheless, this should not be regarded as a limitation of study. Indeed, RF algorithm perfectly depicts a specific patient profile marked by high inflammatory disease and comorbidity burden. In these cases, the prompt start of tocilizumab or methotrexate may surge as a valid therapeutic strategy for preventing flares and disability. In our study, we acknowledge a limited sample size due to the low prevalence of GCA and strict inclusion criteria for the historical cohort. Additionally, only a few patients in our cohort had histopathological confirmation of their GCA, mainly for the unavailability of early multidisciplinary management with vascular surgeons at certain institutions and for the use of temporal artery US as a surrogate of biopsy. Nevertheless, the main limitation of our study is the lack of the standardization of GCs induction and tapering dose due to the retrospective design of the study. Although all tertiary centers relied on former EULAR recommendations for GCs tapering aiming at 10–15 mg/day at three months (2), we may not exclude the difference between tapering regimens for each patient during the observation period. In conclusion, to date, this is the first report evaluating the potential validity of ML algorithm to predict GCA disease flare upon GCs tapering. Our algorithm needs external validation in independent cohorts to provide a reproducible tool available for clinical practice as it must be noticed that recall (sensitivity) remains fairly low for all the three algorithms. Nevertheless, it may already give a glimpse of which GCA patients deserve close monitoring and timely initiation of major immunosuppression.

## Data Availability Statement

The original contributions presented in the study are included in the article/**Supplementary Material**. Further inquiries can be directed to the corresponding author.

## Ethics Statement

The study involving human participants were reviewed and approved by the Ethics Committee of the University of Bari (GISEA registry, IRB approval n. DG-624, Azienda Ospedaliera Universitaria di Bari; ClinicalTrial.Gov NCT01543594). Written informed consent to participate in this study was provided by the participants’ legal guardian/next of kin. The patients/participants provided their written informed consent to participate in this study.

## Author Contributions

VV, FI, and GL conceived the study design, drafted the manuscript, and contributed to discussion. VV performed statistical analysis and drafted the manuscript. GE, LuCa, PL, MF, CF, NL, LaCo, IM, GR, DM, ST, AP, MLU, and BF collected the data and contributed to discussion. All authors listed have made a substantial, direct, and intellectual contribution to the work and approved it for publication.

## Conflict of Interest

The authors declare that the research was conducted in the absence of any commercial or financial relationships that could be construed as a potential conflict of interest.

## Publisher’s Note

All claims expressed in this article are solely those of the authors and do not necessarily represent those of their affiliated organizations, or those of the publisher, the editors and the reviewers. Any product that may be evaluated in this article, or claim that may be made by its manufacturer, is not guaranteed or endorsed by the publisher.
